# Effect of Trace Zn Addition on Interfacial Evolution in Sn-10Bi/Cu Solder Joints during Aging Condition

**DOI:** 10.3390/ma12244240

**Published:** 2019-12-17

**Authors:** Qingfeng Wang, Hong Chen, Fengjiang Wang

**Affiliations:** 1 School of Naval Architecture & Ocean Engineering, Jiangsu University of Science and Technology, Zhenjiang 212003, China; wang76910@163.com; 2 Provincial Key Laboratory of Advanced Welding Technology, Jiangsu University of Science and Technology, Zhenjiang 212003, China; chen15751776962@163.com

**Keywords:** Sn-Bi solder, solid solution, intermetallic compounds, aging, Zn addition

## Abstract

Excessive growth of intermetallic compounds (IMCs) during service affects the reliability of solder joints, so how to suppress the growth of IMC thickness at the interface in solder joints becomes a widespread concern. In this work, the interfacial reaction between Sn-10Bi solder and Cu substrate after thermal aging was investigated. Moreover, to depress the IMC growth at the interface, trace amounts of Zn was added into the Sn-10Bi solder, and the interfacial reactions of Sn-10Bi-xZn solders (x = 0.2, 0.5) and Cu substrate after thermal aging were studied in this paper. Compounds such as Cu_6_(Sn, Zn)_5_ and Cu_5_Zn_8_ were formed at the interface after adding trace amounts of Zn. The addition of 0.2 and 0.5 wt% Zn significantly inhibited the thickness growth of IMCs and the formation of Cu_3_Sn IMC at the interface of Sn-10Bi-0.2Zn/Cu and Sn-10Bi-0.5Zn/Cu during thermal aging. Therefore, the addition of trace Zn had an obvious effect on the interfacial reaction of Sn-10Bi/Cu solder joint. Interestingly, the evolution of IMC thickness in Sn-10Bi-0.5Zn/Cu solder joints was completely different from that in Sn-10Bi or Sn-10Bi-0.2Zn solder joints, in which the spalling of IMCs occurred. In order to explore the mechanisms on the depressing effect from the addition of trace Zn, the activation energy *Q* in solder joints during aging was calculated.

## 1. Introduction

Due to the harmfulness of Pb to the human body and environment, Pb-free solders received an increasing attention in research and application in packaging in recent years [1,2]. At present, Sn-Ag-Cu series solders are the most widely used Pb-free solders for their favorable performances [3]. However, other solders are being developed due to a high Ag cost and lower drop reliability from Sn-Ag-Cu solders [4]. Sn-Bi solder is one of them due to its low melting point (139 °C) and good mechanical properties [5]. However, large amounts of Bi in Sn-58Bi eutectic increases the brittleness of the solder alloy because of the brittleness nature of Bi phases [6]. In Sn-Bi solder, Bi atoms can also exist as a solid solution in the β-Sn phase, which acts as a solid solution strengthening. Therefore, Sn-Bi solders with lower Bi content, especially Sn-Bi solid solution solders provide potential application to replace Sn-Ag-Cu series solders due to their similar melting point [7,8]. Wang et al. [9] investigated the interfacial behavior in Sn-Bi solid solution solder on Cu and found that Sn-Bi solder with low Bi content presented lower growth of intermetallic compound (IMC) thickness and higher joint strength compared with pure Sn solder. Ye et al. [10] studied the mechanical properties of Sn-Bi solder with different Bi content and found that the hardness of Sn-Bi solder with a Bi content of about 10% was the highest among all studied Sn-Bi solder alloys. Lai et al. [11,12] compared Sn-10Bi solder with Sn-Ag-Cu solder, and found that Sn-10Bi solder presented higher creep resistance. Therefore, Sn-10Bi solder provides a possible replacement for Sn-Ag-Cu solder.

It is well known that intermetallic compounds (IMCs) are formed by the reaction of solder with substrate to realize the interconnecting between them during soldering. The IMCs between Sn-based solder and Cu are mainly Cu_6_Sn_5_ and Cu_3_Sn, which will gradually grow and thicken with the service time. Due to the embrittlement of IMCs, solder joints are prone to brittle fracture due to excessive growth of IMCs during service, which deteriorates the reliability of solder joints [13]. Therefore, various methods were used to inhibit the excessive growth of IMCs. At present, doping trace elements is a relatively common and practical method. The effects of doping various elements in solder alloy on the interfacial reaction and reliability of solder joints were studied [14]. It has been found that the addition of trace Zn was very effective in controlling the interfacial reaction with Cu. The formation of Cu_3_Sn IMC, the consumption of Cu, and the formation of interfacial voids were significantly reduced by the addition of Zn [15,16,17]. El-Daly et al. [18,19] explored the effects of Zn addition on microstructure, thermal behavior, and tensile creep properties of Sn-1.0Ag-0.3Cu solder alloy, and Zn addition could improve the tensile strength, ductility and creep resistance. Kang et al. [15] observed that a small amount of Zn added into Sn-Ag-Cu solder can reduce supercooling during solidification, thereby inhibiting the formation of large Ag_3_Sn plates in solder. Mokhtari et al. [20] doped a trace amount of Zn into Sn-58Bi solder and found that the addition of 0.7 wt% Zn significantly inhibited the thickness growth of IMC in Sn-58Bi solder joint under reflow and thermal aging. Adding a small amount of Zn to Sn-0.7Cu solder was studied by Wang et al. and found that it can significantly reduce the formation of Cu_6_Sn_5_, and when the amount of doped Zn exceeded 0.8 wt%, the IMC would become Cu_5_Zn_8_ [21]. Wang et al. also found that the addition of 0.2 wt% Zn to pure Sn solder significantly suppressed the thickness growth of IMCs [22].

In this paper, we used Sn-10Bi solder as the solder matrix, and tried to incorporate trace amounts of Zn into it. The interfacial reaction and atomic migration mechanism of solder/Cu joints during thermal aging were then studied.

## 2. Materials and Methods

The solder alloys used in this experiment were Sn-10Bi, Sn-10Bi-0.2Zn, and Sn-10Bi-0.5Zn, respectively. They were vacuum smelted from pure Sn (99.99%), pure Bi (99.99%), and pure Zn (99.99%) with correct weight percent at 600 °C in vacuum furnace. The substrate was high purity Cu foil with a thickness of 0.3 mm. The copper foils were cleaned with 3% hydrochloric acid to remove the oxide on the surface before use, and then ultrasonically cleaned for 10 min in alcohol. The solder joints were prepared by 0.1g solder balls on Cu foils at 260 °C for 30 s. To investigate the effect of isothermal aging on the interfacial evolution in solder joints, the as-soldered joints were then aged at 130–170 °C for 10–40 days. In order to observe the interfacial microstructure, these samples were cross-sectioned, mounted, metallurgically polished, and then etched with 1% hydrochloric acid for 5 s. Scanning electron microscopy (SEM) was used to observe the interfacial microstructure between solder and Cu. The composition of the IMCs at the interface was analyzed by energy dispersive X-ray (EDX). The thickness of IMCs at the interface was measured with software Photoshop CS6.0 by dividing the IMC area by length of the interface.

## 3. Results

### 3.1. As-Soldered Joints

**Figure 1:**
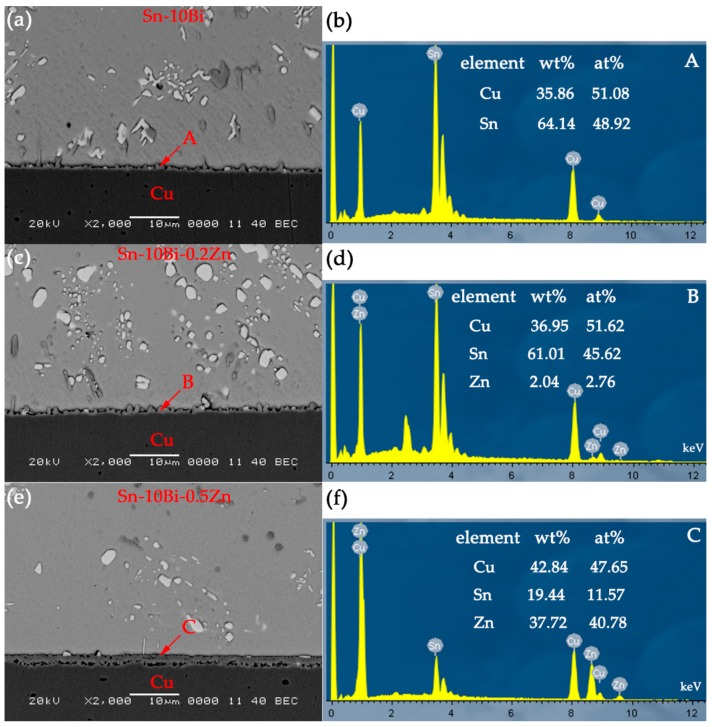
Interfacial structure and the corresponding elemental compositions of interfacial IMC in as-soldered joints: (**a**,**b**) Sn-10Bi/Cu, (**c**,**d**) Sn-10Bi-0.2Zn/Cu, and (**e**,**f**) Sn-10Bi-0.5Zn/Cu.

After Sn-10Bi solder was wetted on Cu substrate, Figure 1a shows the interfacial structure of Sn-10Bi solder on Cu. The top and bottom area represent the solder and Cu substrate, respectively. Meanwhile, a very thin layer of scalloped IMC was formed at the interface between solder and Cu substrate, as shown in Figure 1a. According to the EDX result on point A, the IMC consists of Cu atoms and Sn atoms with the atomic percentage ratio of 6:5, as shown in Figure 1b. Therefore, the IMC formed in Sn-10Bi/Cu solder joint during soldering was Cu_6_Sn_5_, and the thickness thereof is measured to be about 1 μm. The IMC layer was mainly obtained from the reaction between Sn atoms from the solder matrix and Cu atoms from the substrate. Furthermore, during soldering process, some Cu_6_Sn_5_ particles were spalled from IMC layer and segregated inside the solder. With 0.2Zn addition into Sn-10Bi solder, as shown in Figure 1c, the interfacial microstructure of Sn-10Bi-0.2Zn/Cu solder joint after soldering is similar to that of Sn-10Bi/Cu, and the IMC thickness is about 1.05 μm. However, EDX analysis of point B in Figure 1d showed that Zn atoms diffused into IMC to form Cu_6_(Sn, Zn)_5_ together with Cu atoms and Sn atoms at the interface. With 0.5Zn doping into Sn-10Bi solder, the IMC generated at the interface of Sn-10Bi-0.5Zn/Cu is greatly different from the other two. As shown in Figure 1e, the shape of the IMC is a flat layer, and it can be seen that the IMC is composed of two layers, and the color of one near solder is deeper than that near Cu substrate. According to EDX analysis of the dark IMC at point C, as shown in Figure 1f, the ratio of Cu atoms to Zn atoms is close to 1:1, so that the compounds having a darker color near solder matrix is Cu_5_Zn_8_, and the lighter color compound is analyzed by EDX as Cu_6_(Sn, Zn)_5_. At the same time, it can be seen that the generated Cu_6_Sn_5_ does not fill the gap between dark IMC and Cu substrate. From Figure 1a,c,e, the bright white particles in solder matrix are Bi phases. During soldering condition, Bi phases did not participate in the reaction between solder and Cu, but would diffuse towards the interface and eventually accumulate among the scalloped IMCs.

### 3.2. Interfacial Evloution in Sn-10Bi Solder Joints during Isothermal Aging

**Figure 2:**
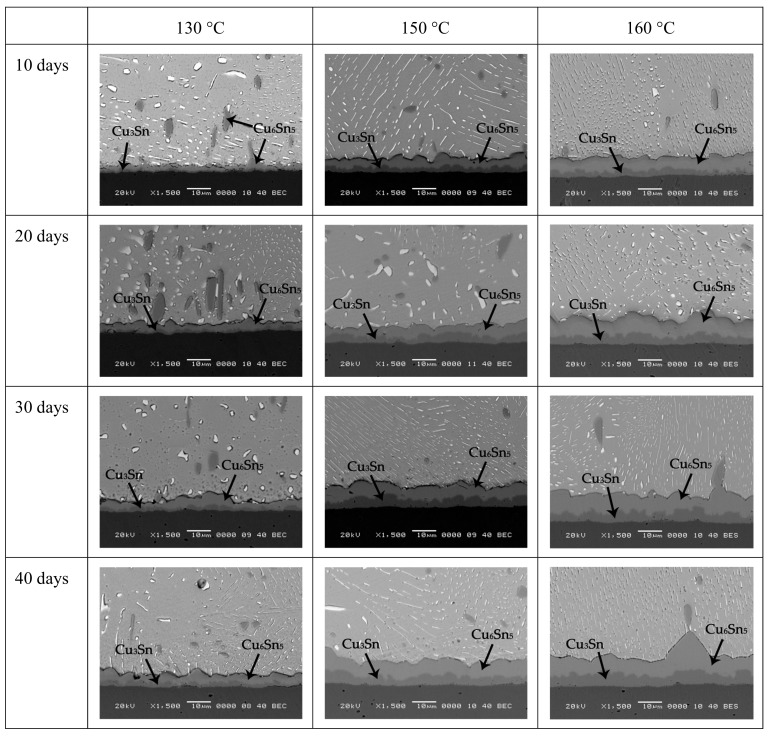
IMC growth in Sn-10Bi/Cu solder joints after thermal aging at different temperatures and times.

Figure 2 shows the interfacial evolution in Sn-10Bi solder joints after isothermal aging. The aging temperature for Sn-10Bi solder was selected as 130, 150, and 160 °C, while for Sn-10Bi-Zn solder was selected as 130, 150, and 170 °C. The temperature of 170 °C was not used for Sn-10Bi solder because the excessive thickness of IMC at the interface caused the joint to be easily broken within a shorter aging time. It can be found that the growth of interfacial IMCs was obviously observed and another Cu_3_Sn IMC was also produced between Cu_6_Sn_5_ and Cu. When the samples aged at 130 °C, the generated Cu_3_Sn was less, and the reaction at the interface was mainly the reaction of Cu atoms with Sn atoms to form Cu_6_Sn_5_. Cu_6_Sn_5_ IMCs were produced in large quantities at the interface over time, and the increase on IMC thickness was mainly attributed to the growth of Cu_6_Sn_5_. When the aging temperature was 150 °C, a distinct Cu_3_Sn layer appeared within 10 days. Cu_3_Sn was obviously generated in the subsequent aging process, and its thickness was almost one-half of the thickness of the entire IMC. Both Cu_6_Sn_5_ and Cu_3_Sn IMC layers grew with the prolonging aging time. With aging temperature increasing to 160 °C, the thickness of IMC layers furtherly increased. Comparing the changes in IMC, it was found that the increase of temperature caused the rate of IMC generation to speed up. From the observation on IMC morphology, the IMC layer flattened after the aging condition. For Bi distribution, Bi phases were transformed into a sheet-like distribution in solder matrix. 

### 3.3. Interfacial Evloution in Sn-10Bi-0.2Zn Solder Joints during Isothermal Aging

**Figure 3:**
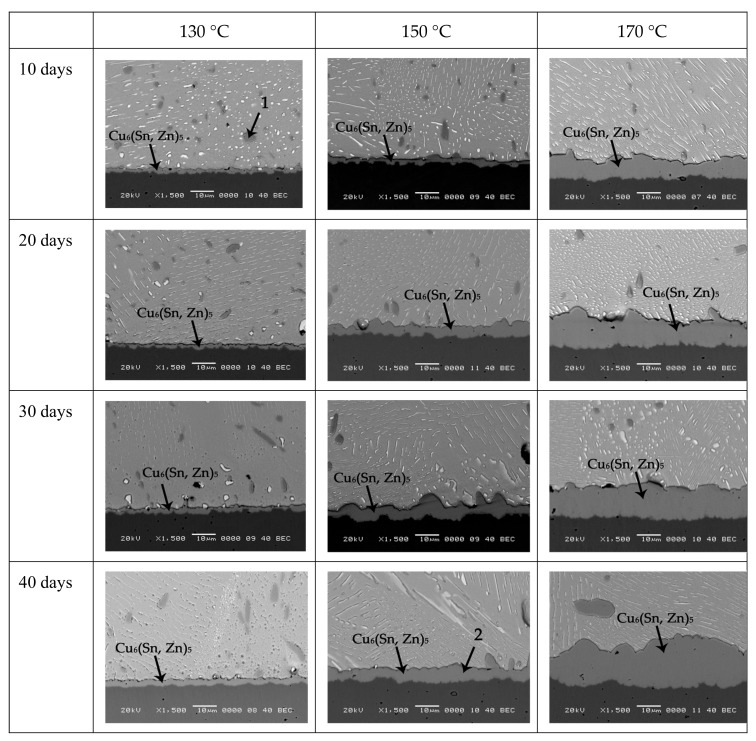
IMC growth of Sn-10Bi-0.2Zn/Cu solder joints after thermal aging at different temperatures and times.

With 0.2Zn addition into the solder, it can be seen from Figure 3 that IMCs at the interface of Sn-10Bi-0.2Zn/Cu also increased during aging, but the growth rate of IMCs obviously decreased. At an aging temperature of 130 °C, the thickness growth of IMCs at the interface is almost negligible. IMCs were thickened with the aging time when the aging temperature increased to 150 °C. At 170 °C, the IMC thickness furtherly increased with the aging time. Compared with Sn-10Bi/Cu solder joints under the same aging temperature and time, the thickness of IMCs of Sn-10Bi-0.2Zn/Cu solder joint obviously reduced. Moreover, unlike the scalloped IMCs at the interface of Sn-10Bi/Cu, IMCs at Sn-10Bi-0.2Zn/Cu became flatter. It is noted that there was no Cu_3_Sn formation at the interface, which was independent of the aging temperature and aging time. The EDX analysis of the phases marked in Figure 3 was detected to confirm their chemical compositions and the corresponding phases are listed in Table 1. It can be seen that only one compound was produced at the interface, and Zn atoms were participated in the interfacial reaction to produce Cu_6_(Sn, Zn)_5_ IMCs at the interface. Therefore, it seems that the depressing effect on IMC growth from 0.2Zn addition into Sn-10Bi solder was mainly attributed to the prevented formation of Cu_3_Sn at the interface of Cu_6_(Sn, Zn)_5_ and Cu. Similarly, Bi phases in solder matrix after isothermal aging presented a scattered particle distribution, and also did not enroll in the interfacial reaction.

### 3.4. Interfacial Evloution in Sn-10Bi-0.5Zn Solder Joints during Isothermal Aging

**Figure 4:**
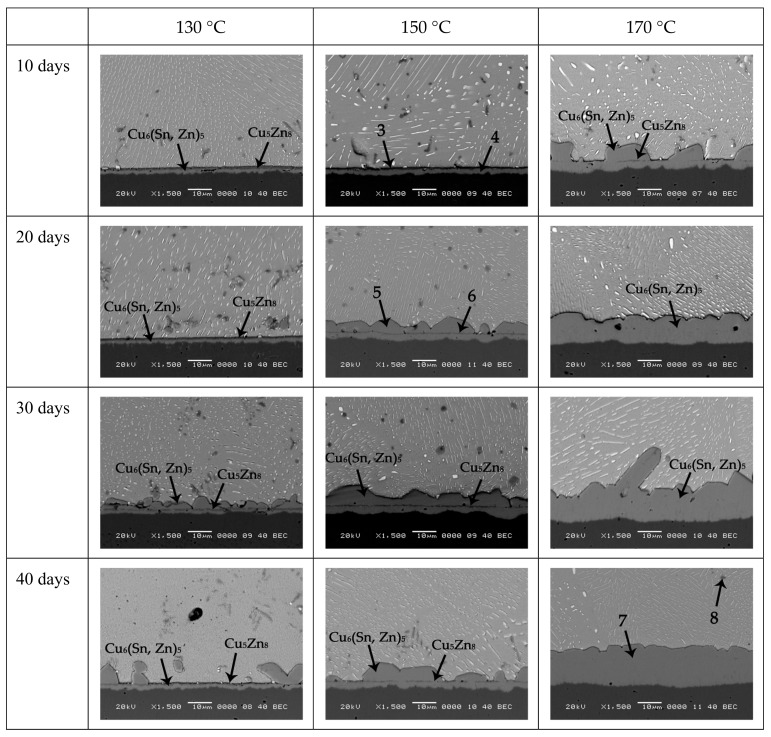
IMC growth of Sn-10Bi-0.5Zn/Cu solder joints after thermal aging at different temperature.

Figure 4 shows the interfacial evolution in Sn-10Bi-0.5Zn solder joints under different aging temperatures and aging times. The thickness of IMCs at the interface of Sn-10Bi-0.5Zn/Cu solder joints which aged at 130 and 150 °C was greatly reduced compared with that of Sn-10Bi/Cu solder joints, and the morphology evolution of IMCs during aging is also completely different from that in Sn-10Bi or Sn-10Bi-0.2Zn solder joints. We can observe three IMC layers at the interface for joints aged at 130 and 150 °C. The corresponding phases in Figure 4 were also detected with XRD with the results listed in Table 1. The phases with dark color at the interface were similar as the result observed in the as-soldered joint and were composed of Cu_5_Zn_8_ IMC. Their morphology was presented as a thin flattened layer. The phases with light gray color underneath the Cu_5_Zn_8_ IMC layer were composed of Cu_6_(Sn, Zn)_5_. The phases located between solder and the Cu_5_Zn_8_ IMC layer were also listed as Cu_6_(Sn, Zn)_5_. Their morphology was presented as a scallop type. At an aging temperature of 170 °C, Cu_5_Zn_8_ can only be observed with aging time of 10 days, while Cu_6_(Sn, Zn)_5_ was the main composition with longer aging times.

As previously mentioned, the IMCs formed in Sn-10Bi-0.5Zn/Cu joints during soldering included two layers: Cu_5_Zn_8_ and Cu_6_Sn_5_. At an aging temperature of 130 °C for 10 and 20 days, layer phenomenon occurred in solder joints, meanwhile, it can be found that the gap between Cu_5_Zn_8_ and Cu substrate was fully filled by Cu_6_(Sn, Zn)_5_. With aging time prolonging to 30 days, the Cu_6_(Sn, Zn)_5_ IMC layer was then produced between the layered Cu_5_Zn_8_ and solder matrix with scallop morphology. After 40 days aging, this scallop type Cu_6_(Sn, Zn)_5_ IMC layer furtherly increased, but it can be found that the scallop Cu_6_(Sn, Zn)_5_ IMC was also spalled into solder matrix to induce a thinner IMC layer. Moreover, the dissolution of interfacial Cu_5_Zn_8_ layer was gradually dissolved with isothermal aging. On the other hand, Cu_3_Sn was also completely depressed by 0.5Zn addition in Sn-10Bi solder at the interface of Cu_6_(Sn, Zn)_5_ IMC and Cu substrate. Therefore, the growth of interfacial IMC layer was mainly contributed by the formation and the spalling effect of Cu_6_(Sn, Zn)_5_ IMC, but would be slower than that in Sn-10Bi solder joints. Moreover, there were two Cu_6_(Sn, Zn)_5_ IMC layers: One with scallop-type distribution between solder matrix and Cu_5_Zn_8_ layer, and the other with flatten-type distribution between Cu_5_Zn_8_ layer and Cu substrate.

After the aging temperature increased to 150 °C, the growth rate of interfacial IMCs was accelerated, and the morphology of IMCs completed the above change within 20 days. Similarly, some scallop-like IMCs disappeared after 40 days of aging due to the spalling effect. With aging temperature increasing, the thickness of two Cu_6_(Sn, Zn)_5_ IMC layers increased compared with the results with an aging temperature of 130 °C. 

When the aging temperature increased to 170 °C, the spalling and dissolution of the Cu_5_Zn_8_ IMC layer was completed within 10 days. With aging time prolonging, the interfacial IMCs were mainly composed of Cu_6_(Sn, Zn)_5_. The growth on IMC thickness was then contributed by Cu_6_(Sn, Zn)_5_ IMC in the subsequent aging.

These changes in the shape of IMCs are actually related to the reaction between intrinsic metal atoms, which we discuss later. Meanwhile, it is worth noting that Cu_3_Sn also did not form when the Sn-10Bi-0.5Zn/Cu solder joints aged at three temperatures.

## 4. Discussion

**Figure 5:**
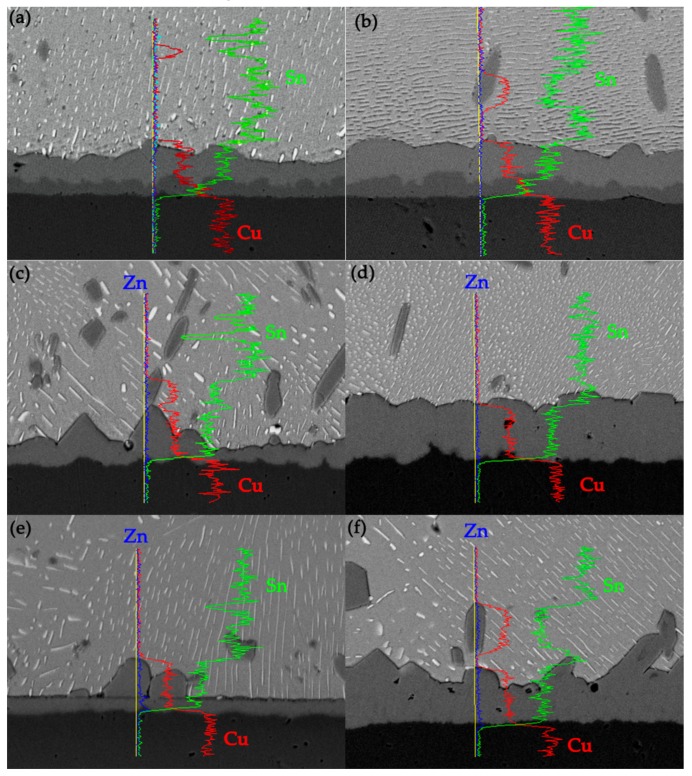
Line scan of interfaces in solder joints: (**a**) Sn-10Bi aged for 40 days at 150 °C, (**b**) Sn-10Bi aged for 30 days at 160 °C, (**c**) Sn-10Bi-0.2Zn aged for 40 days at 150 °C, (**d**) Sn-10Bi-0.2Zn aged or 30 days at 170 °C, (**e**) Sn-10Bi-0.5Zn aged for 40 days at 150 °C, and (**f**) Sn-10Bi-0.5Zn aged for 30 days at 170 °C.

The compositions of interfacial IMCs in Sn-10Bi/Cu joints are Cu_6_Sn_5_ and Cu_3_Sn during isothermal aging. It can be seen from the line scan at the interface in Figure 5a,b that Cu atoms are mainly presented in Cu substrate and IMC layer, and Sn atoms are mainly presented inside the solder matrix and IMC layer. Bi atoms are distributed in the solder matrix and do not enter the IMC layer and Cu substrate. With Zn doped into solder, the elemental composition at the interface changed. The atomic percentage at point 2 in Table 1 is 60.11% for Cu atoms, 36.62% for Sn atoms, and 3.27% for Zn atoms. Therefore, Zn atoms in the Sn-10Bi-0.2Zn solder diffused into IMC and replaced some Sn atoms to form Cu_6_(Sn, Zn)_5_ [23]. Since these Zn atoms in IMC hindered the diffusion of Cu atoms into solder and Sn atoms towards substrate, they inhibited the formation and growth of the Cu_6_(Sn, Zn)_5_ IMC layer. By performing a line scan at the interface of Sn-10Bi-0.2Zn/Cu solder joints aged at 150 °C for 40 days and 170 °C for 30 days, the content of Zn atoms in the IMC range obviously increased, which indicates that Zn atoms in Sn-10Bi-0.2Zn solder were dissolved into IMC, as shown in Figure 5c,d. As reported in a previous paper [24], Zn atoms were easily segregated at the interface between Cu substrate and IMC layer to form CuZn solid solution with Cu atoms, but it was not easily to be observed at the interface in Figure 5c.

With more Zn addition, Zn atoms in Sn-10Bi-0.5Zn solder continuously diffused from the solder matrix to the interface and combined with Cu atoms to form Cu_5_Zn_8_ IMCs. Since the Gibbs free energy (∆*G*) of Cu_5_Zn_8_ phase (−12.34 kJ/mol) was much lower than that of Cu_6_Sn_5_ phase (−7.42 kJ/mol) [25], Zn atoms were more reactive than Sn atoms with Cu atoms, and accordingly the formation of Cu_5_Zn_8_ was superior to Cu_6_Sn_5_.Therefore, in Figure 1e, Cu atoms preferentially reacted with Zn atoms to form Cu_5_Zn_8_ at a distance from the Cu substrate, and then reacted with Sn atoms to form Cu_6_Sn_5_ between Cu_5_Zn_8_ and Cu substrate. Since the reaction was insufficient within a shorter soldering time, the generated Cu_6_Sn_5_ could not fill the gap between Cu_5_Zn_8_ and Cu substrate. A significant gap existed between Cu_5_Zn_8_ and Cu substrate. After aging with a shorter time, two IMC layers were also observed at the interface. It was found by EDX analysis that the compound near the solder matrix was Cu_5_Zn_8_, and the compound near Cu substrate was Cu_6_ (Sn, Zn)_5_, as shown at point 3 and 4. Moreover, the thickness of Cu_6_(Sn, Zn)_5_ layer increased with aging temperature and time, which indicates that during aging, Sn and Zn atoms were continuously migrated towards the interface and reacted with Cu atoms to form Cu_6_(Sn, Zn)_5_ IMCs. Liu et al. [26] also reported the evolution of the metastable Cu_5_Zn_8_ phase at the interface of Sn-3.7Ag-0.9Zn/Cu joints. After 1 min of soldering, the metastable Cn_5_Zn_8_ phase was formed. Then, part of the Cu_5_Zn_8_ layer was transformed into the stable Cu_6_Sn_5_ phase near Cu plate. With aging time increasing, according to EDX analysis, the compound at point 5 is Cu_6_(Sn, Zn)_5_, and the thin layered compound at point 6 is Cu_5_Zn_8_. Therefore, due to the instability of Cu_5_Zn_8_ at high temperature [27], Cu_5_Zn_8_ gradually decomposed into Zn atoms and Cu atoms during aging. Cu atoms combined with Sn atoms to form a more stable Cu_6_Sn_5_, while Zn atoms entered the continuously generated Cu_6_Sn_5_ to form Cu_6_(Sn, Zn)_5_ [26]. Therefore, the interface in 0.5Zn contained solder joints after aging at 130 and 150 °C was composed of Cu_6_(Sn, Zn)_5_, Cu_5_Zn_8_, and Cu_6_(Sn, Zn)_5_. After aging at 170 °C, the Cu_5_Zn_8_ IMC layer was completely decomposed within a short aging time, and the interface was mainly composed of Cu_6_(Sn, Zn)_5_. 

A line scan of interface of Sn-10Bi-0.5Zn/Cu joints aged at 150 °C for 40 days showed that there were two peaks in the content of Zn atoms, as shown in Figure 5e: One at the linear compounds in IMC and the other at the interface of IMC and Cu. For solder joint aged at 170 °C, as shown in Figure 5f, the Cu_5_Zn_8_ compounds disappeared, and the content of Zn atoms in Cu_5_Zn_8_ compounds that diffused into solder became remarkable, which indicates that Cu_5_Zn_8_ continuously decomposed with the increase of aging time. The instability of Cu_5_Zn_8_ also affected the IMC between Cu_5_Zn_8_ and solder. It can be inferred that accompanying with the decomposition of Cu_5_Zn_8_, Cu_6_Sn_5_ layer between Cu_5_Zn_8_ and solder partly detached from IMC layer according to the special IMC morphology in joints aged at 130 and 150 °C for longer time. In Figure 5c,e,f, the peaks of Zn atoms content appear at the interface between Cu and IMC, indicating that Zn atoms diffused into Cu substrate to form Cu_5_Zn_8_ after continuous aging. Moreover, the presence of Zn atoms in Cu_6_(Sn, Zn)_5_ prevented the reaction between Cu substrate and Cu_6_(Sn, Zn)_5_ to form Cn_3_Sn between them.

Since the growth of IMC at solder/Cu during solid aging is volume diffusion-controlled, it is well known that it follows the classical diffusion equation:

(1) $$X=X_{0}+\sqrt{Dt}$$

where *X* represents the IMC thickness, *X*_0_ represents the initial IMC thickness, *D* is the diffusivity, and *t* is the aging time. The slope of the fitted line, $\sqrt{D}$, represents the growth rate of IMC. 

**Figure 6:**
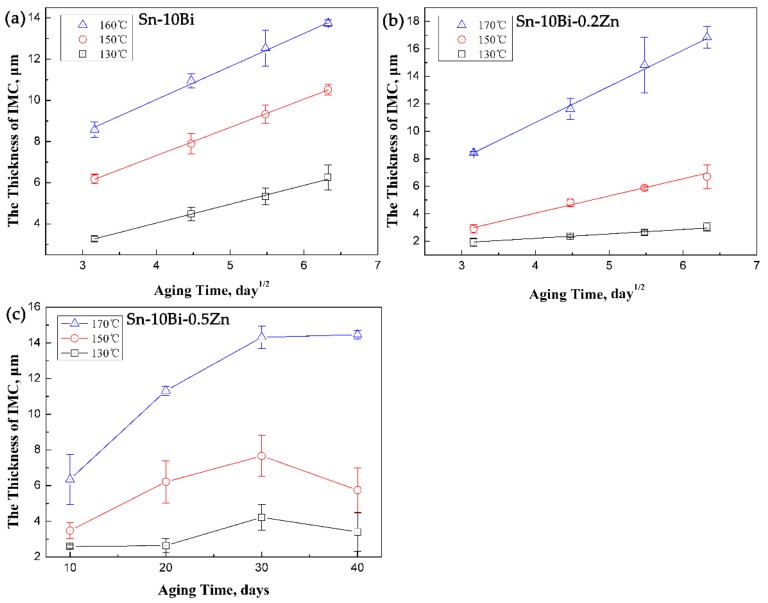
Effect of isothermal aging on IMC growth occurred at the interface of (**a**) Sn-10Bi/Cu, (**b**) Sn-10Bi-0.2Zn/Cu, and (**c**) Sn-10Bi-0.5Zn/Cu.

Figure 6a,b are results on the thickness of IMCs in Sn-10Bi/Cu and Sn-10Bi-0.2Zn/Cu solder joints at different aging temperatures and aging times. The linear fit confirms that the thickness growth of IMCs in Sn-10Bi/Cu and Sn-10Bi-0.2Zn/Cu solder joints is proportional to *t*^0.5^. 

Figure 6c is the thickness of IMCs in Sn-10Bi-0.5Zn/Cu solder joints after aging at different temperatures. It is obvious that the thickness growth of IMC in Sn-10Bi-0.5Zn/Cu solder joints did not follow the classical diffusion formula because of the formation and dissolution of Cu_5_Zn_8_ layer and the spalling effect of Cu_6_(Sn, Zn)_5_ IMC layer.

According to the data in Figure 6a, the interfacial IMCs in Sn-10Bi/Cu solder joints continues to grow during isothermal aging, and its growth rate is accelerated with aging temperature increasing. The growth rate of the interfacial IMCs in Sn-10Bi/Cu solder joints is 0.915 μm/day^1/2^, 1.370 μm/day^1/2^, and 1.605 μm/day^1/2^ under 130, 150, and 160 °C, respectively. Therefore, an obvious difference on them with the effect of aging temperature does not exist. However, the addition of 0.2Zn obviously affects the growth rate of interfacial IMC layer. It can be seen from Figure 6b that the growth rate of IMCs at the interface of Sn-10Bi-0.2Zn/Cu under aging temperature of 130 and 150 °C is evidently reduced, and the influence of aging temperature on the growth rate is accelerated. Comparing Figure 6a with Figure 6b, it is found that the thickness of IMCs in Sn-10Bi joints grew from 3.2 to 6.3 μm at 130 °C, while that in Sn-10Bi-0.2Zn solder joints grew from 1.9 to 2.8 μm. The growth rate of IMCs of Sn-10Bi-0.2Zn solder joints is much lower than that of Sn-10Bi joints, indicating that the addition of 0.2Zn greatly inhibited the reaction of Sn atoms in the solder with Cu at 130 °C. However, the inhibition effect is deteriorated at higher aging temperature. For example, the IMCs in Sn-10Bi solder joints aged at 150 °C grew from 6. 2 to 11 μm, while the IMCs of Sn-10Bi-0.2Zn grew from 2.9 to 6.3 μm. Accordingly, the IMCs growth rate of Sn-10Bi and Sn-10Bi-0.2Zn solder joints is 1.370 and 1.252 μm/day^1/2^, respectively. Overall, it is clearly seen that the diffusion rate increased as the aging temperature increased.

It is obvious that the growth of IMCs in Sn-10Bi-0.5Zn did not follow the classical diffusion formula. In Figure 6c, during aging at 130 °C for 10 or 20 days, the overall growth of IMC was very slow due to the simultaneous reaction of Cu atoms with Zn and Sn atoms. With aging period prolonging to 30 days, the IMC grew rapidly because the reaction between Sn and Cu atoms became the dominant reaction. After 30 days aging, Cu_6_(Sn, Zn)_5_ was produced between Cu_5_Zn_8_ and solder matrix, which induced an increase on IMC thickness. With aging days of 40 days, the spalling effect of Cu_6_(Sn, Zn)_5_ was faster than its formation, which induced a decrease on IMC thickness. It should be noted that the decomposition of Cu_5_Zn_8_ always occurred, which affected the formation of Cu_6_Sn_5_ and Cu_3_Sn between Cu_5_Zn_8_ and solder. At an aging temperature of 150 °C, the reaction was accelerated due to the increase on atomic diffusion. The reaction between Sn and Cu atoms became the dominant reaction during 10 to 30 days, and the IMC continued to grow during this period. The thickness of IMC also decreased after 30 days due to the spalling effect of Cu_6_(Sn, Zn)_5_. When the temperature increased to 170 ° C, the reaction was furtherly accelerated. Combined with Figure 5, it is found that the formation and decomposition of Cu_5_Zn_8_ completed within 10 days aging. As shown in Figure 5, during aging from 10 days to 40 days, Zn atoms diffused into the solder and Cu matrix, while Sn atoms continuously reacted with Cu atoms to induce the growth of IMCs.

In order to explore the reason why the growth of IMC is slowed after the addition of 0.2Zn, the activation energy *Q* of the solder is calculated. In Fick’s first law, the diffusion coefficient *D* can be expressed by:

(2) $$D=D_{0}e\frac{-Q}{RT}$$

where *D*_0_ is the diffusion constant, *R* is the general gas constant, *T* is the absolute temperature, and *Q* is the activation energy. This formula can be rewritten as:

(3) $$\ln D=\ln D_{0}-\frac{Q}{RT}$$

Therefore, in the curve with 1/*T* as the *x*-axis and lnD as the *y*-axis, the slope of the curve indicates the activation energy *Q*. The greater the activation energy is, the smaller the diffusion coefficient is, and the slower the IMC growth is. The diffusion coefficient *D* and aging temperature *T* of the growth of IMCs in Sn-10Bi/Cu and Sn-10Bi-0.2Zn/Cu solder joints are listed in Table 2. 

**Figure 7:**
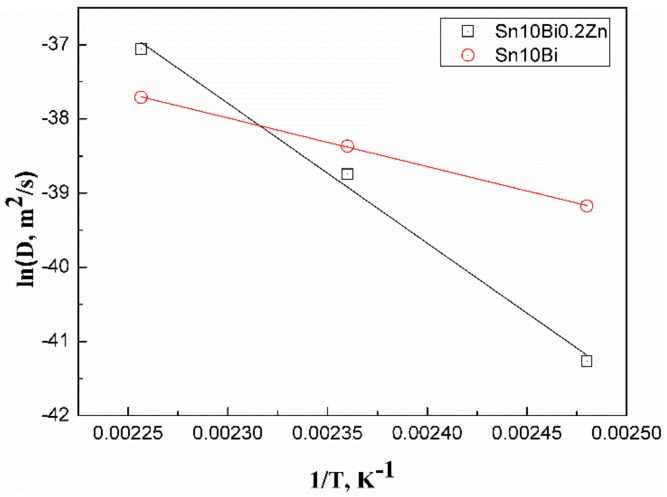
Arrhenius plots of IMC layer formed at the interface of Sn-10Bi/Cu and Sn-10Bi-0.2Zn/Cu.

The Arrhenius plots are drawn from the relationship between aging temperature and diffusion coefficient with the results shown in Figure 7. Accordingly, the activation energy *Q* can be calculated. The activation energy of IMCs growth of Sn-10Bi/Cu is 54.61 kJ/mol, and that of Sn-10Bi-0.2Zn/Cu is 157.19 kJ/mol. The activation energy of IMCs growth of Sn-10Bi-0.2Zn/Cu is much higher than that of Sn-10Bi/Cu. Therefore, the energy required for the interfacial reaction of the Sn-10Bi-0.2Zn/Cu joint is much higher than that of Sn-10Bi/Cu joint, and the formation rate of IMC is also slowed down.

**Figure 8:**
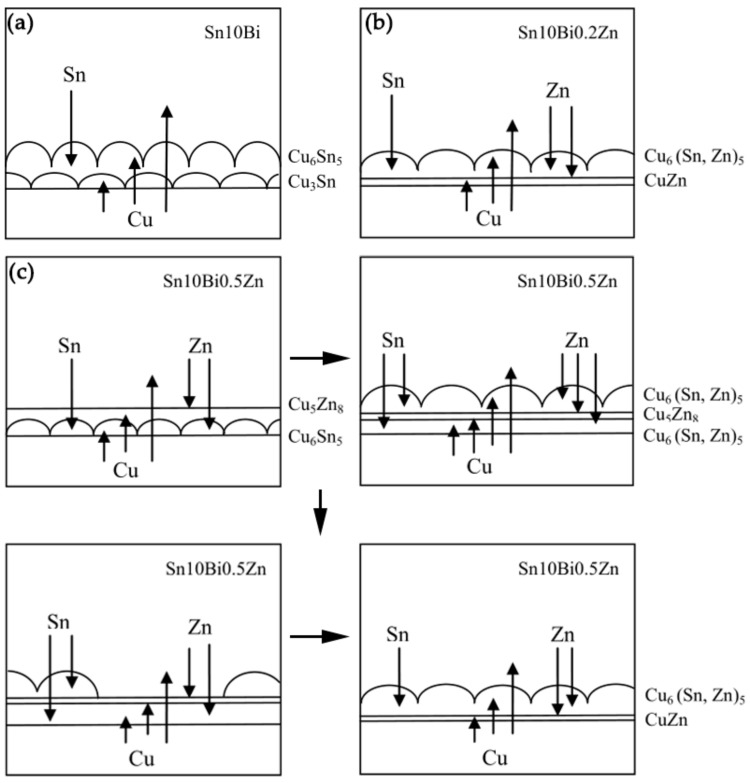
Schematic diagrams on atomic diffusion and reaction at the interface occurred in (**a**) Sn-10Bi, (**b**) Sn-10Bi-0.2Zn, and (**c**) Sn-10Bi-0.5Zn solder joints during isothermal aging.

Furtherly, the intrinsic atomic migration and interaction during aging were investigated. Figure 8 shows the atomic diffusion and reaction at the interface during isothermal aging. After the temperature increased, the atoms became active and diffused to each other. In Sn-10Bi/Cu solder joints shown in Figure 8a, Bi atoms were mainly dissolved in β-Sn phase, and did not enroll in the reaction. Sn and Cu atoms were the main diffusing atoms. During aging, Sn atoms continuously diffused from solder to Cu substrate and Cu atoms diffused from substrate to the solder side. After reaction, Cu_6_Sn_5_ was continuously formed at the interface between solder and Cu substrate, as shown in Equation (4). Therefore, the Cu_6_Sn_5_ IMC layer formed during soldering continuously grew with aging.

6Cu + 5Sn → Cu_6_Sn_5_ (4)

Moreover, Cu_3_Sn was also formed at the interface between Cu_6_Sn_5_ and Cu substrate due to the reaction of the diffused Cu atoms with Cu_6_Sn_5_, as shown in the Equation (5).

9Cu + Cu_6_Sn_5_ → 5Cu_3_Sn (5)

It can be seen that Cu_6_Sn_5_ was formed at the interface between solder and IMC and Cu_3_Sn was formed at the interface between Cu substrate and IMC. This indicates that the growth of Cu_6_Sn_5_ was mainly controlled by the diffusion of Sn atoms, while the growth of Cu_3_Sn was mainly controlled by the diffusion of Cu atoms. The formation of Cu_6_Sn_5_ and Cu_3_Sn led to a continuous increase on IMC thickness at the interface.

After the addition of 0.2% Zn, the change on the interfacial reaction occurred. Zn atoms in the solder did not react with Sn atoms, but continuously diffused towards the interface and Cu_6_Sn_5_ IMCs. As shown in the EDX of point 9, Zn atoms diffused into the Cu_6_Sn_5_ crystal structure and replaced some Sn atoms to form Cu_6_(Sn, Zn)_5_. At the same time, Zn atoms in IMCs hindered the inter diffusion of Sn atoms and Cu atoms, thus suppressing the formation of Cu_6_Sn_5_. Since the activation energy of Cu_5_Zn_8_ is much smaller than that of Cu_3_Sn, there was no Cu_3_Sn production at the interface. With time increasing, the solubility of Zn atoms in Cu_6_Sn_5_ was saturated. Some Zn atoms diffused into Cu substrate to form the CuZn solid solution, and some were enriched at the interface between IMCs and Cu substrate. The enrichment of Zn atoms and the CuZn solid solution became diffusion barriers, hindering the diffusion of Cu atoms and Sn atoms and suppressing the formation of IMCs. Hence, the growth of IMC of Sn-10Bi-0.2Zn/Cu was much slower than that of Sn-10Bi/Cu, as shown in Figure 8b.

It is possible that the content of Zn atoms in Sn-10Bi-0.2Zn/Cu solder joint was too small, and no obvious Cu_5_Zn_8_ compound was observed at the interface. However, the product at the interface changed when the content of Zn atoms increased to 0.5%. Firstly, Zn atoms diffused towards Cu substrate and reacted with Cu atoms to form Cu_5_Zn_8_, as shown in Equation (6).

5Cu + 8Zn → Cu_5_Zn_8_ (6)

Secondly, Cu atoms reacted with the diffused Sn atoms to form Cu_6_Sn_5_ between Cu_5_Zn_8_ and Cu substrate. Then, some Zn atoms diffused into the Cu_6_Sn_5_. As shown in the initial period of aging at 130 and 150 °C in Figure 4, the top layer of the IMC had a flatter appearance. Thirdly, Cu atoms diffused into the solder to react with Sn atoms. Cu_6_Sn_5_ formed at the interface between solder and Cu_5_Zn_8_, and a small amount of Zn atoms were still diffused therein. At the same time, the decomposition of Cu_5_Zn_8_ began to occur due to its instability, as shown in Equation (7).

Cu_5_Zn_8_ → 5Cu + 8Zn (7)

Part of the decomposed Cu atoms and Zn atoms diffused towards Cu_6_Sn_5_ between Cu_5_Zn_8_ and Cu substrate, while some of them diffused into the solder side. Simultaneously, Cu_6_(Sn, Zn)_5_ was also produced between the Cu_5_Zn_8_ layer and solder matrix. However, due to the poor adhered effect of Cu_6_(Sn, Zn)_5_ on Cu_5_Zn_8_, a part of Cu_6_Sn_5_ was easily separated from IMCs. After the decomposition of Cu_5_Zn_8_, part of Zn atoms diffused toward Cu substrate and were concentrated at the interface between IMCs and Cu substrate. Zn atoms that diffused into Cu substrate were mainly dissolved in the Cu atoms to form a small layer of CuZn solid solution. The interfacial evolution in Sn-10Bi-0.5Zn joints was then plotted in Figure 8c.

## 5. Conclusions

The effect of trace Zn addition on the interfacial reaction and the growth of IMCs of Sn-10Bi solder on Cu joints under different aging temperatures were explored in this study. The following conclusions can be drawn:

(1) Both 0.2% and 0.5% Zn addition into Sn-10Bi solder can effectively depress the growth of interfacial IMCs in Sn-10Bi solder joints during isothermal aging, but they presented different mechanisms on the depressing effect.
(2) With 0.2% Zn addition, Zn atoms can be dissolved into the interfacial IMCs to form Cu_6_(Sn, Zn)_5_. Zn atoms were segregated along the interface between Cu-Sn IMCs and Cu substrate, which completely inhibited the formation of Cu_3_Sn between Cu_6_(Sn, Zn)_5_ and Cu. The lower IMC growth rate was mainly attributed to the deficiency of Cu_3_Sn and higher activation energy of IMCs growth.
(3) With 0.5% Zn addition, Zn atoms firstly reacted with Cu atoms to form Cu_5_Zn_8_, which played as a diffusion barrier layer to inhibit the inter diffusion of Sn and Cu atoms. At lower aging temperature, the interface was composed of Cu_6_(Sn, Zn)_5_, Cu_5_Zn_8_, and Cu_6_(Sn, Zn)_5_. Moreover, Cu_6_(Sn, Zn)_5_ IMCs formed on Cu_5_Zn_8_ was easily spalled into the solder matrix to induce the decrease on IMCs thickness. At higher aging temperature, the metastable Cu_5_Zn_8_ was decomposed and the interface was only composed of Cu_6_(Sn, Zn)_5_.

## Figures and Tables

**Table 1:** EDX analysis on the elemental components at various points in figures.

Point	Chemical Composition (at%)			Phase
	Cu	Sn	Zn	
1	51.86	48.14	0.0	η-Cu_6_Sn_5_
2	60.11	36.62	3.27	Cu_6_(Sn, Zn)_5_
3	46.20	15.47	38.33	γ-Cu_5_Zn_8_
4	52.74	41.85	5.42	Cu_6_(Sn, Zn)_5_
5	51.68	44.09	4.23	Cu_6_(Sn, Zn)_5_
6	52.93	20.43	26.64	γ-Cu_5_Zn_8_
7	52.76	44.27	2.97	Cu_6_(Sn, Zn)_5_
8	41.41	16.97	41.62	CuZn

**Table 2:** Diffusion rate and activation energy during interfacial reaction of different solders.

Solder	Temperature (°C)	*D* (m^2^·s^−1^)	*Q* (kJ·mol^−1^)
Sn-10Bi	130	9.69 × 10^−18^	54.61
	150	21.72 × 10^−18^	
	160	29.82 × 10^−18^	
Sn-10Bi-0.2Zn	130	1.20 × 10^−18^	157.19
	150	18.14 × 10^−18^	
	170	80.12 × 10^−18^	
